# Lesson learned from the investigation and response of Lassa fever outbreak, Margibi County, Liberia, 2018: case report

**DOI:** 10.1186/s12879-019-4257-z

**Published:** 2019-07-11

**Authors:** Abyot Bekele Woyessa, Leroy Maximore, Darius Keller, John Dogba, Myer Pajibo, Kumblytee Johnson, Emmanuel Saydee, Julius Monday, Roland Tuopileyi, Nuha Mahmoud

**Affiliations:** 1 World Health Organization Country Office for Liberia, Monrovia, Liberia; 2 Margibi County Health Team, Kakata, Liberia; 3 National Public Health Institute of Liberia, Monrovia, Liberia; 4 CH Rennie Hospital, Kakata, Liberia; 5 Kakata District Health Team, Kakata, Liberia

**Keywords:** Lassa fever, Viral hemorrhagic diseases, Contact case, Outbreak, Case report, Lesson learned, West Africa, Liberia

## Abstract

**Background:**

Lassa fever (LF) is a viral hemorrhagic disease caused by the Lassa virus (LASV) and endemic in West African countries with an estimation of 300,000 to 500,000 cases and 5,000 deaths annually. The Margibi County Health Team of Liberia received a report of an unidentified febrile illness case from the Kakata district. We conducted the investigation to identify the causative agent and the source of infection to support treatment, control and prevention interventions.

**Case presentation:**

We identified LASV in the blood specimens’ of two patients by Reverse Transcriptase Polymerase Chain Reaction (RT-PCR). Both the confirmed cases have manifested respiratory distress, weakness, and difficulty of swallowing, muscle, joint and back pains, and vomiting with blood. The symptoms started with mild fever and gradually developed. Initially, the primary health facilities have miss-diagnosed the patients as malaria and respiratory tract infections. The primary health facilities have referred the patients to the referral hospital as the patients have failed to respond to antimalarial and antibiotics. The hospital suspected LF and sent blood specimens to the National Reference Laboratory while the patients were on supportive treatment in the isolation room. At the time when the laboratory result returned to the hospital, the patients died of LF illness before ribavirin administered.

**Conclusions:**

Our investigation revealed that the two hospitalized and deceased febrile cases were associated with LASV. The primary health facilities have failed to recognize the cases as suspected LF at the earliest time possible. The clinicians and health facilities, especially primary health facilities, need to consider LF as a differential diagnosis when the patient failed to respond to anti-malaria and broad-spectrum antibiotics.

## Background

Lassa fever (LF) is a zoonotic disease and potentially deadly hemorrhagic illness caused by Lassa Virus (LASV), a single-stranded RNA and a member of the *Arenaviridae* family [1], first described in West Africa in the 1950s [2]. However, the LASV was isolated, recognized and named in 1969 from a missionary nurse who worked in a clinic in a small town, Lassa, in Northeastern Nigeria [3, 4]. The multimammate mouse, *Mastomysnatalensis*, is the primary host species for LASV and widely distributed throughout West, Central, and East African countries [5–7]. LASV is a highly virulent and contagious viral infection [8]. Rodent-to-human transmission of LASV occurs via contact with rodent’s body fluids, excreta, urine, tissues, or blood, as well as inhalation of infectious aerosols [9]. Additionally, direct or indirect contact with the blood, urine, faeces, or other bodily secretions of infected person appears to be the route often involved in the transmission of LASV from person to person [10]. All age groups are susceptible and possibly affected by LASV [11].

The symptoms of LF are often protean and nonspecific [12] and clinical diagnosis is very difficult as the signs and symptoms are indistinguishable from diseases that are common in the tropics, such as severe malaria, typhoid fever, yellow fever and other viral hemorrhagic fevers [13]. The incubation period of LF is usually around 10 days (6–21 days) after exposure to the virus [14, 15]. Person-to-person transmission may theoretically occur during the acute febrile phase and an infected person may excrete the virus in urine for 3–9 weeks from the onset of illness [16].

The disease is endemic in West African countries, including Nigeria, Serra Leone, Liberia and Guinea [17, 18]. Irrespective of the evidence of its transmission, the seroprevalence of LASV is believed to be lower in Cote d’Ivoire, Ghana, Togo, and Benin [19]. Ghana reported the first domestic LF cases in 2011 [20] while two imported cases were also reported in 2015 [21]. From 1969 to 2015, over 30 imported cases of LF were reported among travelers from West African countries in the United States, Germany, United Kingdom, Netherlands, Israel, Canada, Japan and Sweden [22, 23]. Secondary infections or local transmissions did not occur in countries reported imported cases [24].

About 80% of each Sierra Leone and Liberia, 50% of Guinea, 40% of Nigeria, 30% of each of Coˆte d’Ivoire, Togo, and Benin, and 10% of Ghana geographical areas were estimated as LF risk area [25]. The LF is endemic in the West African countries and causes 300,000 to 500,000 cases annually with about 5,000 deaths [26]. In some areas of Sierra Leone and Liberia, 10–16% of annual hospital admissions are due to LF [18].

In Liberia, the magnitude of LF mortality and morbidity is underreported [27]. However, the disease remains a major public health challenge and its endemicity gained an expanded area in the country [27, 28]. The prevalence of antibody to LASV ranges from 15 to 20% in Liberia [8]. The Ministry of Health of the Republic of Liberia has been reported LF outbreak and sporadic cases in different parts of the country. For instance, in February 2016; 14 confirmed cases with 29% CFR were reported [29]. A high prevalence of LASV was also documented among hospital health workers in Liberia and around the border of Serra Leone [30].

In April 2017, Margibi County Health Team of Liberia received a report of an unidentified febrile illness case, from CH Rennie hospital through Kakata District Health Team. The Margibi County Health Team commenced an investigation to identify the causative agent and source of infection to support treatment, control, and prevention interventions as part of the routine public health emergency management operations.

## Case presentation

### Case 1

On April 26, 2018, a 56-year-old teacher from Jambo village of Kakata City admitted to the local clinic with high fever, diarrhea, abdominal, muscles and joint pains, loss of appetite and difficulty of swallowing. He traveled to Bong County a week before his illness to attend his relative’s funeral ceremony who died of an unknown illness. After he returned from the funeral ceremony, he became sick on April 25, 2018. On the following day, just 1 day after the onset of the symptoms, he sought medical care and visited the clinic. The clinicians at the clinic treated and discharged him home with some antibiotics and anti-pain medicines on the same day he visited the health facility. He stayed at his home from April 27, 2018, to May 2, 2018, while using the prescribed drugs. However, the illness gradually aggravated and his family brought him back to the clinic on May 3, 2018. After checking his vital signs, the clinic referred him to the referral hospital with the diagnosis of respiratory tract infection (RTI). When he reached to the referral hospital, his clinical signs and symptoms worsen, and he started manifesting new symptoms including vomiting with blood and respiratory distress. The hospital performed a malaria test by a microscope, but no parasite detected in his blood. The hospital admitted the patient in an isolation room; and sent a blood specimen to the National Reference Laboratory at the National Public Health Institute of Liberia (NPHIL) on May 4, 2018. However, the patient died of the illness on May 4, 2018, at around 4–5:00 am before the laboratory results returned. The National Reference Laboratory has tested the specimen for Ebola virus disease and the result turned negative. Furthermore, they have tested the specimen by RT-PCR and detected LASV RNA. The hospital received the laboratory result from the reference laboratory through the County Health Team at 5:00 pm on May 4, 2018, after the dead body provided to the family.

### Case 2

A 43-year-old pregnant woman (16 weeks) from Meana Town of Gibi District was sick on April 23, 2018. She had a mild fever at the beginning of her illness. She did not seek medical attention as early as possible considering that her illness was mild. New sign and symptoms gradually appeared and the illness was getting worse. She started manifesting respiratory distress, weakness, and difficulty of swallowing, muscle, joint and back pains. Following the appearance of the new symptoms, she sought medical care at one of the public clinics in Peter Town on May 2, 2018. The clinic treated her for malaria in pregnancy and prescribed quinine, amoxicillin and paracetamol tablets. They referred her to another health facility, private clinic, on May 3, 2018, where she admitted for two nights. Moreover, the clinic later transferred her to the referral hospital on May 5, 2018. The referral hospital admitted her to obstetrics and gynecology ward and gave her supportive treatment. However, her clinical condition was not improved and she started vomiting with blood. At this point, the clinician at the hospital suspected LF and sent a blood specimen to the National Reference Laboratory on May 7, 2018, and the result turned positive for LASV. However, the patient died of her illness before the hospital able to provide ribavirin, which is available and effective antivirus for LF treatment.

Four ml blood specimens were collected from the two acute febrile cases in a lavender top K_3_EDTA glass tube and transported to the National Reference Laboratory at NPHIL. The specimens were first tested for Ebola virus disease (EVD) and then tested for LASV by RT-PCR which is a gold standard diagnosis for LASV [31] to detect RNA virus. Previously established and described Trombley RT-PCR assay was used to test specimens for LASV [32].

**Fig. 1:**
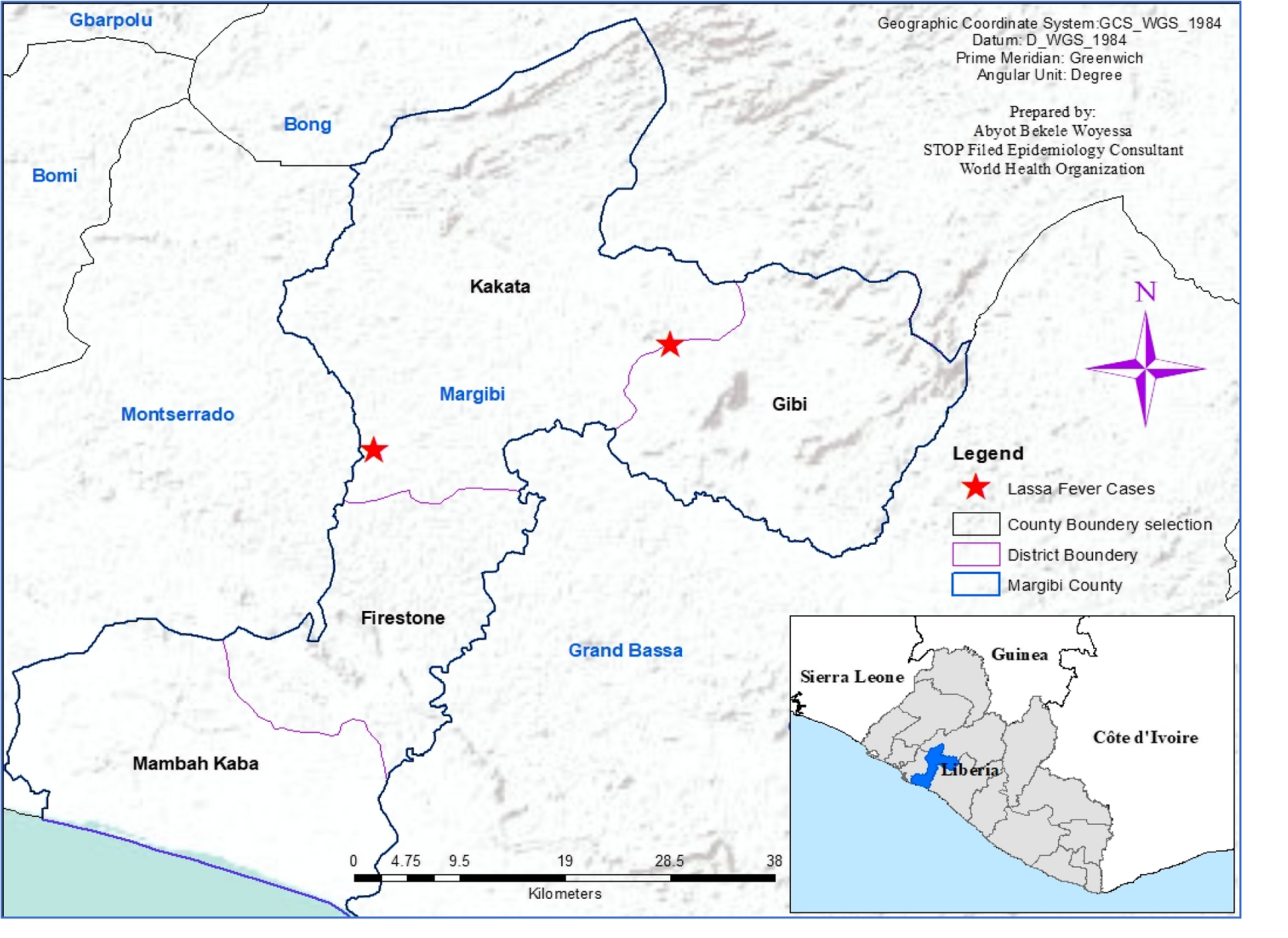
Map showing the distribution of confirmed Lassa fever cases, Margibi County, Liberia, May 2018

We captured the geographic coordinates of residential places of the two cases using a mobile-based global positioning system (GPS) and created a point map on the base map of Liberia, using Arc GIS 10.4.1 (Fig. 1).

Following the detection of the first case, we strengthened the public health surveillance system at a district, health facility, and community levels through distributing standard and community case definitions and increasing awareness. We conducted an active case search in all health facilities and communities through engaging health workers and community informants. Accordingly, we identified five LF alert cases, cases with unexplained fever and ruled out for malaria, through an active case search. A blood specimen was collected from all the reported alert cases. The hospital admitted the alert cases in the isolation room and provided supportive treatment while laboratory results waited. We ruled out all the five alert cases, as the specimens from all patients were negative for LASV by RT-PCR at the national reference laboratory.

The Margibi Health Team together with its partners worked to trace and identify family members, health workers, and friends who may have had contact with the patients or their body fluids or their dead bodies. Contact tracers recruited, trained and deployed to identify all contacts. Through discussions with the family members, caregivers, and health workers, the contact tracers have identified 89 contacts and categorized them to low and high-risk contacts based on the level and degree of their contact with confirmed patients. Twenty-two (25%) and 67 (75%) contacts were classified as high and low-risk contacts respectively. Among the total contacts, 31 (35%) had contact with first case while 58 (65%) had contact with second case. Most of the contacts, 32 (36%) were health care workers (HCW). All the contacts have been followed-up by trained contact tracers for 21 days after the last date of contact with the case. All the contacts have completed the follow-up period. We did not observe an associated case among contacts.

Following the detection of the LASV from the blood specimen of the two diseased patients, we reinforced infection prevention and control (IPC) measures in all public and private health care facilities. We sensitized and mentored the health workers on LF prevention, use of appropriate Personal Protective equipment (PPE), hand hygiene practices and waste management procedures. Furthermore, we updated, printed, and distributed the LF flyers and brochures to health workers and community informants. The county health promoter has presented LF jingles at two radio stations in the county. The county health team has activated the emergency operations center, and coordinated the response interventions on a daily bases.

Trained and protected morticians of the hospital placed the dead body of the first LASV confirmed case in a waterproof plastic body bag in an isolation room and taken to the morgue. Moreover, the morticians and country surveillance officer placed the body on the car and taken directly to the funeral home on May 4, 2018, at 11:00 am. The county surveillance officer advised the family members, relatives, and friends not to touch the dead body while viewing to avoid the possible risk of infections. In addition, the surveillance officer supervised the dead body at the funeral home. The county burial team escorted and transported the dead body from the funeral home to the burial site to ensure safe and dignified burial practice on May 05, 2018 at 12:00 pm.

Similarly, the morticians managed the dead body of the second case and placed it in a plastic body bag. We kept and supervised the body in the morgue room of the hospital until the laboratory result returned back. The family members prepared a burial place while the dead body was in the morgue of the hospital. Ensuring the readiness of the burial place, the burial team placed the dead body on a car and took it directly to the burial site in Gibi district. The county burial team managed dignified and safe burial procedures. The hospital disinfected the morgue room by chlorine solutions after the dead body taken to the burial site.

## Discussion and conclusions

The investigation uncovered that the cause of the two deaths from febrile illness was associated with LASV. Our report highlights the difficulty of timely detecting and diagnosis of LF at primary health facilities. The non-specific clinical pictures of LF usually make clinical diagnosis difficult in the absence of laboratory confirmed index case. In the two cases described here, the treatment of the patient for other diseases obviously delayed the consideration of other endemic diseases like LF as a potential cause of the febrile illness. The majority of contacts were comprising of health workers which is similar to the study conducted in Nigeria [33].

The second case had no known contact with confirmed and/or suspected cases of LF. She did not travel to other areas in 3 weeks’ time prior to her illness. She might have been infected through direct contact with infected rodents or indirectly contacted with the urine and dropping of the infected rodents in or at around her house.

The cause of death of the two described LF cases might be attribute to the late referral and detection of the cases. The first confirmed case has died of the virus after 10 days of the onset of the first sign and symptoms of the illness and before the laboratory result returned. Even though the patient went to a health facility the earliest time after the onset of a fever, the health workers did not suspect LF. This might be attributed to non-specific clinical picture of the disease at the beginning [12, 34]. In contrast to the first case, the second case visited a health facility for medical care lately or after 10 days of the first onset of the illness. However, the co-infection of malaria may be the reason that the clinician at the local health facility failed to suspect LF as the earliest time possible. Malaria co-infections and miss-diagnosis also observed in other several studies. In addition to delaying at home and miss-diagnoses, referring the patient from health facility to health facility delays laboratory investigation in which the patient died before the laboratory result was known. Because of late laboratory confirmation, the hospital failed to treat the cases for LF with an appropriate antiviral. The study conducted in Serra Leone indicated that among untreated laboratory LASV confirmed cases, 87% had a chance of death [35] and the CFR was less among patients treated with ribavirin [36].

Our investigation and response to the two confirmed cases had some limitations. The cause of death for the young girl in Bong County was not traced and investigated, at which the first case attended the funeral ceremony a week before his illness, which might be the source of his infection. In January 2018, there were two suspected cases and one RT-PCR confirmed LF death reported from Bong county [37, 38]. In addition, in the investigation we could not managed to conduct an environmental investigation through accessing the availability of rodents and their holes and droppings. There is also a public health action gaps on the management and burial procedures of the first case as the hospital provided the dead body to the family before the cause of the illness not known.

Our investigation revealed that the underlying causative agent for the two patients died of febrile illness was associated with LASV. The two deceased cases have reported from different places and there was no epidemiological linkage between the cases. Late referral and laboratory confirmation led to miss-diagnosis, which further led to death because of the absence of early specific treatment. The source of infection needs to be identified through conducting an environmental assessment to prevent future possible outbreaks through engaging communities’ in discouraging a rodent to enter the house, though keeping the house clean, covering food and making it inaccessible to the rodents and promoting hygienic conditions.

In conclusion, both the public and private health facilities should consistently implement standard infection prevention and control interventions when caring for febrile patients to prevent nosocomial infections. As LF is clinically nonspecific and co-infection of malaria may be common in the region, health facilities and clinicians should consider LF and or other viral diseases as a differential diagnosis if the patient failed to respond to anti-malaria and/or broad-spectrum antibiotics. We also believe that robust syndromic surveillance for LF and other viral hemorrhagic diseases is significantly important in endemic areas to timely detect the cases. The specimen should have timely collected and sent to the national reference laboratory for early confirmation to support the triage system at health facilities, contact tracing and as well as for initiation of treatment with ribavirin to reduce the possible high case fatality rate.

## Data Availability

The datasets used to prepare this manuscript are presented here and accessible from Margibi County Health Team and from the corresponding author anytime on reasonable request.

## Abbreviations

CFR: Case Fatality Rate
EVD: Ebola Virus Disease
GIS: Geographic Information System
HCW: Health Care Worker
IPC: Infection prevention and control
LASV: Lassa Virus
LF: Lassa fever
NPHIL: National Public Health Institute of Liberia
RNA: Ribonucleic acid
RTI: Respiratory tract infection
RT-PCR: Real-Time Polymerase Chain Reaction
